# KwARG: parsimonious reconstruction of ancestral recombination graphs with recurrent mutation

**DOI:** 10.1093/bioinformatics/btab351

**Published:** 2021-05-10

**Authors:** Anastasia Ignatieva, Rune B Lyngsø, Paul A Jenkins, Jotun Hein

**Affiliations:** Department of Statistics, University of Warwick, Coventry CV4 7AL, UK; Department of Statistics, University of Oxford, Oxford OX1 3LB, UK; Department of Statistics, University of Warwick, Coventry CV4 7AL, UK; Department of Computer Science, University of Warwick, Coventry CV4 7AL, UK; The Alan Turing Institute, British Library, London NW1 2DB, UK; Department of Statistics, University of Oxford, Oxford OX1 3LB, UK; The Alan Turing Institute, British Library, London NW1 2DB, UK

## Abstract

**Motivation:**

The reconstruction of possible histories given a sample of genetic data in the presence of recombination and recurrent mutation is a challenging problem, but can provide key insights into the evolution of a population. We present KwARG, which implements a parsimony-based greedy heuristic algorithm for finding plausible genealogical histories (ancestral recombination graphs) that are minimal or near-minimal in the number of posited recombination and mutation events.

**Results:**

Given an input dataset of aligned sequences, KwARG outputs a list of possible candidate solutions, each comprising a list of mutation and recombination events that could have generated the dataset; the relative proportion of recombinations and recurrent mutations in a solution can be controlled via specifying a set of ‘cost’ parameters. We demonstrate that the algorithm performs well when compared against existing methods.

**Availability and implementation:**

The software is available at https://github.com/a-ignatieva/kwarg.

**Supplementary information:**

Supplementary data are available at *Bioinformatics* online.

## 1 Introduction

For many species, the evolution of genetic variation within a population is driven by the processes of mutation and recombination in addition to genetic drift. A typical mutation affects the genome at a single position, and may or may not spread through subsequent generations by inheritance. Recombination, on the other hand, occurs when a new haplotype is created as a mixture of genetic material from two different sources, which can drive evolution at a much faster rate. The detection of recombination is an important problem that can provide crucial scientific insights, for instance in understanding the potential for rapid changes in pathogenic properties within viral populations (Simon-Loriere and Holmes, 2011).

**Fig. 1.**
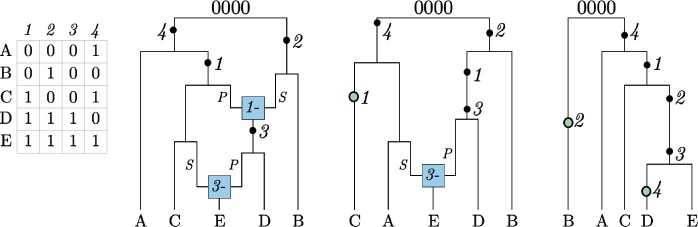
Three examples of ARGs. The dataset is shown on the left in binary format, with 0’s and 1’s corresponding to the ancestral and mutant state at each site, respectively. Mutation events are shown as black dots and labelled by the site they affect; green filled circle corresponds to a recurrent mutation. Recombination nodes (in blue) are labelled with the recombination breakpoint; material to the right (left) of the breakpoint is inherited from the parent connected by the edge labelled *S* (*P*) for ‘suffix’ (‘prefix’)

Consider a population evolving through the replication, mutation and recombination of genetic material within individuals, emerging from a common origin and living through multiple generations until the present day. In general, the history of shared ancestry, mutation and recombination events are not observed, and must be inferred from a sample of genetic data obtained from the present-day population. Crossover recombination can occur anywhere along a sequence, and the breakpoint position is also unobserved. This article focuses on methods for reconstructing possible histories of such a sample, in the form of *ancestral recombination graphs* (*ARGs*)—networks of evolution connecting the sampled individuals to shared ancestors in the past through coalescence, mutation and crossover recombination events; an example is illustrated in Figure 1. This is a very important but challenging problem, as many possible histories might have generated a given sample. Moreover, recombination can be undetectable unless mutations appear on specific branches of the genealogy (Hein *et al.*, 2004, Section 5.11), and recombination events can produce patterns in the data that are indistinguishable from the effects of *recurrent mutation* (McVean *et al.*, 2002); that is, two or more mutation events in a genealogical history that affect the same locus.

Parsimony is an approach focused on finding possible histories which minimize the number of recombinations and recurrent mutations. This does not necessarily describe the most biologically plausible version of events, but produces a useful lower bound on the complexity of the evolutionary pathway that might have generated the given dataset. Beyond specifying the types of events that are allowed, parsimony does not require assuming a particular generative model; the approach focuses on sequences of events that can generate the observed dataset, disregarding the timing and prior rate of these events.

Previous work on reconstructing histories using parsimony has tackled recombination and recurrent mutation separately. Algorithms for reconstructing minimal ARGs generally make the *infinite sites assumption*, which allows at most one mutation to have occurred at each site of the genome, thus precluding recurrent mutation events, and the goal is to calculate the minimum number of crossover recombinations required to explain a dataset, denoted *R*_min_. Even with this constraint, the problem is NP-hard (Wang *et al.*, 2001); exact algorithms are practical only for small datasets (Hein, 1990; Lyngsø *et al.*, 2005), and general methods rely on heuristic approximations (Hein, 1993; Minichiello and Durbin, 2006; Parida *et al.*, 2008; Song *et al.*, 2005; Thao and Vinh, 2019). Alternatively, one can assume the absence of recombination and seek to calculate the minimum number of recurrent mutations required, denoted *P*_min_. In this case, reconstruction of maximum parsimony trees is also NP-hard (Foulds and Graham, 1982); likewise, methods can only handle small datasets or are based on heuristics (Semple and Steel, 2003, Section 5.4).

Parsimony contrasts with the alternative approach of model-based inference, which requires the user to select a generative model and relies on the estimation of mutation and recombination rates as model parameters. Model-based inference generally involves integrating over the space of possible histories, which is usually intractable; methods rely on MCMC (e.g. Rasmussen *et al.*, 2014) or importance sampling (e.g. Jenkins and Griffiths, 2011), but the problem remains computationally difficult. If the presence of recombination is certain and reasonable models of population dynamics are available, model-based approaches may be more suitable and result in more powerful inference. However, model misspecification can play an important role, for instance when modelling viral evolution over a transmission network, where the relative importance of factors such as geographical structure, social clustering and the impact of interventions may be difficult to ascertain. In this case, model-based inference can provide misleading results if overinterpreted, with poor quantification of uncertainty due to model misspecification. Parsimony-based methods fail to offer the interpretability or uncertainty quantification of a model but this does preclude their results being overinterpreted. They are simple and straightforward to implement and can be useful in situations such as enabling testing for the presence or absence of recombination when this is not certain (Bruen *et al.*, 2006).

There are a number of recently developed methods, namely RENT+ (Mirzaei and Wu, 2017), tsinfer (Kelleher *et al.*, 2019) and Relate (Speidel *et al.*, 2019), that seek to reconstruct local tree or ARG topologies from the data. These methods do not make strict model-based assumptions, incorporating heuristic algorithms, and do not aim to reconstruct the most *parsimonious* histories. We note also the existence of numerous other methods for inference of recombination (e.g. Boni *et al.*, 2007; Kosakovsky Pond *et al.*, 2006; Li and Stephens, 2003; Martin and Rybicki, 2000) which do not explicitly reconstruct ARGs.

KwARG (‘quick ARG’) is a software tool, written in C, which implements a greedy heuristic-based parsimony algorithm for reconstructing histories that are minimal or near-minimal in the number of posited recombination and mutation events. The algorithm starts with the input dataset and generates plausible histories backwards in time, adding coalescence, mutation, recombination and recurrent mutation events to reduce the dataset until the common ancestor is reached. By tuning a set of cost parameters for each event type, KwARG can find solutions consisting only of recombinations (giving an upper bound on *R*_min_), only of recurrent mutations (giving an upper bound on *P*_min_), or a combination of both event types. KwARG handles both the ‘infinite sites’ and ‘maximum parsimony’ scenarios, as well as interpolating between these two cases by allowing recombinations as well as recurrent mutations and sequencing errors, which is not offered by existing methods. This is illustrated in Figure 1: KwARG finds all three types of solution for the given dataset. KwARG shows excellent performance when benchmarked against exact methods on small datasets, and outperforms existing parsimony-based heuristic methods on large, more complex datasets while maintaining computational efficiency; KwARG also achieves very good accuracy in reconstructing local tree topologies. The source code and executables are made freely available on GitHub at https://github.com/a-ignatieva/kwarg, along with documentation and usage examples.

The article is structured as follows. Details of the algorithm underlying KwARG are given in Section 2, with an explanation of the required inputs and expected outputs. In Section 3, the performance of KwARG on simulated data is benchmarked against exact methods and existing programs. An application of KwARG to a widely studied *Drosophila melanogaster* dataset (Kreitman, 1983) is described in Section 4. Discussion follows in Section 5.

## 2 Materials and Methods

Consider a sample of genetic data, where the allele at each site can be denoted 0 or 1. We do not make the infinite sites assumption, so that each site can undergo multiple mutation events. However, we do assume that mutations correspond to transitions between exactly two possible states, excluding for instance triallelic sites.

### 2.1 Input

KwARG accepts data in the form of a binary matrix, or a multiple alignment in nucleotide or amino acid format. The sequence and site labels can be provided if desired. It is possible to specify a root sequence, or leave this to be determined. The presence of missing data are permitted; regardless of the type of input, the data are converted to a binary matrix D, with entries ‘⋆’ denoting missing entries or material that is not ancestral to the sample.

### 2.2 Methods

Under the infinite sites assumption, at most one mutation is allowed to have occurred per site. If any two columns contain all four of the configurations 00, 01, 10, 11, then the data could not have been generated only through replication and mutation, and there must have been at least one recombination event between the two corresponding sites. This is the four gamete test (Hudson and Kaplan, 1985), and the two sites are said to be *incompatible*. When recurrent mutations are allowed, the incompatibility could likewise have been generated through multiple mutations affecting the same site (McVean *et al.*, 2002).

KwARG reconstructs the history of a sample backwards in time, by starting with the data matrix D and performing row and column operations corresponding to coalescence, mutation and recombination events, until only one ancestral sequence remains. By reversing the order of the steps, a forward-in-time history is obtained, showing how the population evolved from the ancestor to the present sample. When a choice can be made between multiple possible events, a neighbourhood of candidate ancestral states is constructed, using the same general method as that employed in the program Beagle (Lyngsø *et al.*, 2005). A backwards-in-time approach has also been implemented in the programs SHRUB (Song *et al.*, 2005), Margarita (Minichiello and Durbin, 2006) and GAMARG (Thao and Vinh, 2019), all of which adopt the infinite sites assumption but use different criteria for choosing amongst possible recombination events.

#### 2.2.1 Construction of a history

For convenience, assume that the all-zero sequence is specified as the root, and 0 (1) entries of D correspond to ancestral (mutated) sites. Suppose $D_{t}$ is the data matrix obtained after *t − *1 iterations of the algorithm. At the beginning of the *t*th step, KwARG first reduces $D_{t}$, by repeatedly applying the ‘Clean’ algorithm (Song and Hein, 2003) through:

- deleting uninformative columns (consisting of all 0’s);
- deleting columns containing only one 1 (corresponding to ‘undoing’ a mutation present in only one sequence);
- deleting a row if it agrees with another row (corresponding to a coalescence event);
- deleting a column if it agrees with an adjacent column.

Two rows (columns) *agree* if they are equal at all positions where both rows (columns) contain ancestral material, and the sites (sequences) carrying ancestral material in one are a subset of the sites (sequences) carrying ancestral material in the other.

A run of the ‘Clean’ algorithm repeatedly applies these steps to $D_{t}$, terminating when no further reduction is possible. Suppose the resulting data matrix is ${\bar{D}}_{t}$. KwARG then constructs a neighbourhood $N_{t}$ of candidate next states, each one obtained through one of the following operations:

- Pick a row and split it into two at a possible recombination point. Only a subset of possible recombining sequences and breakpoints needs to be considered; see Lyngsø *et al.* (2005, Section 3.3) for a detailed explanation.
- Remove a recurrent mutation, by selecting a column and changing a 0 entry to 1, or a 1 entry to 0. This is the event type that is disallowed by algorithms applying the infinite sites assumption.

Suppose a neighbourhood $N_{t}=\{N_{t}^{1},\;\ldots ,N_{t}^{N}\}$ is formed, consisting of all possible states that can be reached from ${\bar{D}}_{t}$ through applying one of these operations. Then the reduced neighbourhood ${\bar{N}}_{t}=\{{\bar{N}}_{t}^{1},\;\ldots ,{\bar{N}}_{t}^{N}\}$ is formed by applying ‘Clean’ to each state in turn. Each state ${\bar{N}}_{t}^{i}$ is then assigned a score $S({\bar{N}}_{t}^{i},N_{t}^{i},{\bar{D}}_{t})$, combining (i) the cost $C\left(N_{t}^{i},{\bar{D}}_{t}\right)$, defined below, of reaching the configuration $N_{t}^{i}$ from ${\bar{D}}_{t}$, (ii) a measure $\text{AM}\left({\bar{N}}_{t}^{i}\right)$ of the complexity of the resulting data matrix ${\bar{N}}_{t}^{i}$ and (iii) a lower bound $L({\bar{N}}_{t}^{i})$ on the remaining number of recombination and recurrent mutation events still required to reach the ancestral sequence from ${\bar{N}}_{t}^{i}$. Finally, a state is selected, say ${\bar{N}}_{t}^{j}$, based on its score, and we set $D_{t+1}={\bar{N}}_{t}^{j}$. The process of reducing the dataset followed by constructing a neighbourhood and choosing the best move is repeated, until all incompatibilities are resolved and the root sequence is reached. Pseudocode for the ‘Clean’ algorithm and KwARG is given in Supplementary Section S1.

**Fig. 2.**
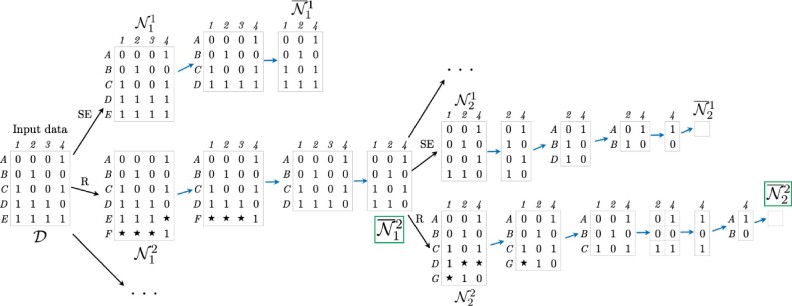
Example of a reconstructed history for the dataset in Figure 1. Stars ‘⋆’ denote non-ancestral material. SE: recurrent mutation occurring on a terminal branch of the ARG. R: recombination event. A sequence of blue arrows corresponds to one application of the ‘Clean’ algorithm. Green boxes highlight the selected states

The construction of a history for the dataset given in Figure 1 is illustrated in Figure 2. The first step corresponds to the construction of a neighbourhood, two of the states $N_{1}^{1},N_{1}^{2}\in N_{1}$ are pictured. Then, the ‘Clean’ algorithm is applied to each state in the neighbourhood (illustrated as a series of steps following blue arrows). From the resulting reduced neighbourhood $\left\{{\bar{N}}_{1}^{1},{\bar{N}}_{1}^{2},\;\ldots \right\}$, the state ${\bar{N}}_{1}^{2}$ is selected; the other illustrated path is abandoned. This process is repeated until all incompatibilities are resolved and the empty state is reached. Following the path of selected moves in this figure left-to-right corresponds to the events encountered when traversing the leftmost ARG in Figure 1 from the bottom up. If instead the state ${\bar{N}}_{2}^{1}$ were selected at the second step of the algorithm, the resulting path would correspond to the ARG in the centre of Figure 1.

#### 2.2.2 Score

When considering which next step to take, more informed choices can be made by considering not just the cost of the step, but also the complexity of the configuration it leads to. This is the principle behind the A* algorithm (Hart *et al.*, 1968), using a heuristic estimate of remaining distance to guide the choice of the next node to expand. KwARG applies the same principle in a greedy fashion, following a path of locally optimal choices in an attempt to find a minimal history.

The score implemented in KwARG is

(1) $$S\left({\bar{N}}_{t}^{i},N_{t}^{i},{\bar{D}}_{t}\right)=\left(C\left(N_{t}^{i},{\bar{D}}_{t}\right)+L\left({\bar{N}}_{t}^{i}\right)\right)\cdot \text{maxAM}\left({\bar{N}}_{t}\right)+\text{AM}\left({\bar{N}}_{t}^{i}\right),$$

where

$$L({\bar{N}}_{t}^{i})=\{\begin{array}{cc} R_{\text{min}}\left({\bar{N}}_{t}^{i}\right) & \text{if}\;\text{maxAM}({\bar{N}}_{t})<75,\\ HB\left({\bar{N}}_{t}^{i}\right) & \text{if}\;75\leq \text{maxAM}({\bar{N}}_{t})<200,\\ HK\left({\bar{N}}_{t}^{i}\right) & \text{otherwise}. \end{array}$$

Here, $C\left(N_{t}^{i},{\bar{D}}_{t}\right)$ denotes the cost of the corresponding event, defined in Section 2.2.3; $\text{maxAM}({\bar{N}}_{t})$ denotes the maximum amount of ancestral material seen in any of the states in ${\bar{N}}_{t}$, and $\text{AM}({\bar{N}}_{t}^{i})$ gives the amount of ancestral material in state ${\bar{N}}_{t}^{i}$. Incorporating a measure of the amount of ancestral material in a state helps to break ties by assigning a smaller score to simpler configurations.

The method of computing the lower bound *L* depends on the complexity of the dataset, with a trade-off between accuracy and computational cost. For relatively small datasets, it is feasible to compute *R*_min_ exactly using Beagle. *HB* refers to the haplotype bound, employing the improvements afforded by first calculating local bounds for incompatible intervals, and applying a composition method to obtain a global bound (Myers and Griffiths, 2003). *HK* refers to the Hudson–Kaplan bound (Hudson and Kaplan, 1985); this is quick but less accurate, so is reserved for larger, more complex configurations. Note that these bounds are computed under the infinite sites assumption.

The particular form and components of the score were chosen through simulation testing; we found that the given formula provides a good level of informativeness regarding the quality of a possible state.

#### 2.2.3 Event cost

Each type of event is assigned a cost, which gives a relative measure of preference for each event type in the reconstructed history:

- *C_R_*: the cost of a single recombination event, defaults to 1.
- *C*_RR_: the cost of performing two successive recombinations, defaults to 2. It is sufficient to consider at most two consecutive recombination events before a coalescence (Lyngsø *et al.*, 2005); this type of event also captures the effects of gene conversion.
- *C*_RM_: the cost of a recurrent mutation. If $N_{t}^{i}$ is formed from ${\bar{D}}_{t}$ by a recurrent mutation in a column representing *k* agreeing sites, this corresponds to proposing *k* recurrent mutation events, so the cost is $C(N_{t}^{i},{\bar{D}}_{t})=k\cdot C_{RM}$.
- *C*_SE_: this event is a recurrent mutation which affects only one sequence in the original dataset, i.e. it occurs on the terminal branches of the ARG. Thus, the event can be either a regular recurrent mutation or an artefact due to sequencing errors. The cost can be set to equal *C*_RM_, or lower if the presence of sequencing errors is considered likely.

KwARG allows the specification of a range of event costs as tuning parameters, as well as the number *Q* of independent runs of the algorithm to perform for each cost configuration. The proportions of recombinations to recurrent mutations in the solutions produced by KwARG can be controlled by varying the ratio of costs for the corresponding event types.

#### 2.2.4 Selection probability

The method of selecting the next state from a neighbourhood of candidates will impact on the efficiency and performance of the algorithm. At one extreme, selecting at random amongst the states will mean that the solution space is explored more fully, but will be prohibitively inefficient in terms of the number of runs needed to find a near-optimal solution. On the other hand, always greedily selecting the move with the minimal score will quickly identify a small set of solutions for each cost configuration, at the expense of placing our faith in the ability of the score to assess the quality of the candidate states accurately.

We propose a selection method that is intermediate between these two extremes, randomizing the selection but focusing on moves with near-minimal scores. A pseudo-score for state ${\bar{N}}_{t}^{i}$ is calculated:

(2) $$\text{exp}(T\cdot \left(1-\tilde{S}\left({\bar{N}}_{t}^{i},N_{t}^{i},{\bar{D}}_{t}\right)\right)),$$

where

$$\tilde{S}\left({\bar{N}}_{t}^{i},N_{t}^{i},{\bar{D}}_{t}\right)=\frac{S\left({\bar{N}}_{t}^{i},N_{t}^{i},{\bar{D}}_{t}\right)-\min_{j}S\left({\bar{N}}_{t}^{j},N_{t}^{j},{\bar{D}}_{t}\right)}{\max_{j}S\left({\bar{N}}_{t}^{j},N_{t}^{j},{\bar{D}}_{t}\right)-\min_{j}S\left({\bar{N}}_{t}^{j},N_{t}^{j},{\bar{D}}_{t}\right)},$$

and states in $\bar{N_{t}}$ are selected with probability proportional to their pseudo-score. The annealing parameter *T* controls the extent of random exploration; *T *=* *0 corresponds to choosing uniformly at random from the neighbourhood of candidates, and T=∞ to always choosing a state with the minimal score. The default value of *T *=* *30 was chosen following simulation testing, which showed that this provides a good balance between efficiency and thorough exploration of the neighbourhood.

### 2.3 Output

The default output consists of the number of recombinations and recurrent mutations in each identified solution; an example for the Kreitman dataset is given in Table 1. Each iteration is assigned a unique random seed, which can be used to reconstruct each particular solution and produce more detailed outputs, such as a detailed list of events in the history, the ARG in several graph formats or the corresponding sequence of marginal trees.

**Table 1.** Example output of KwARG for the Kreitman dataset

Seed	*T*	*C*_SE_	*C*_RM_	*C_R_*	*C*_RR_	SE	RM	R	$\sum_{t}|N_{t}|$
2263536315	30.0	∞	∞	1.00	2.00	0	0	7	143
2347021759	30.0	0.90	0.91	1.00	2.00	1	0	6	853
1791455164	30.0	0.80	0.81	1.00	2.00	1	0	5	728
1684879495	30.0	0.60	0.61	1.00	2.00	2	0	4	783
1884182000	30.0	0.40	0.41	1.00	2.00	3	0	3	806
1900122424	30.0	0.20	0.21	1.00	2.00	5	0	2	702
2111915557	30.0	0.10	0.11	1.00	2.00	8	0	1	833
2888657821	30.0	0.01	0.02	1.00	2.00	10	0	0	715

*Note*: SE: number of recurrent mutations occurring on terminal branches of the ARG (possible sequencing errors); RM: number of other recurrent mutations; R: number of recombinations. Last column gives the total number of neighbourhood states considered.

## 3 Results

We have tested the performance of KwARG on simulated data, based on two main criteria. First, we compared its performance against exact methods, PAUP* and Beagle, to demonstrate that KwARG successfully reconstructs minimal histories in the mutation-only and recombination-only cases, respectively. Second, we carried out simulation studies to determine how accurately KwARG reconstructs local trees, compared against three other methods: tsinfer, RENT+ and ARGweaver. Finally, we compared how well KwARG performs against the parsimony-based heuristic methods SHRUB (Song *et al.*, 2005) and SHRUB-GC (Song *et al.*, 2006); these results are presented in Supplementary Section S4. We also investigated the dependence of the run time of KwARG on the number and length of sequences, through simulation studies.

### 3.1 Finite sites

#### 3.1.1 Comparison to PAUP*

Disallowing recombination, the quality of computed upper bounds on *P*_min_ was tested by comparison with PAUP* (Swofford, 2001, version 4.0a168), which was used to compute the exact minimum parsimony score via branch-and-bound on 994 datasets simulated as described in Supplementary Section S3.1.

**Fig. 3.**
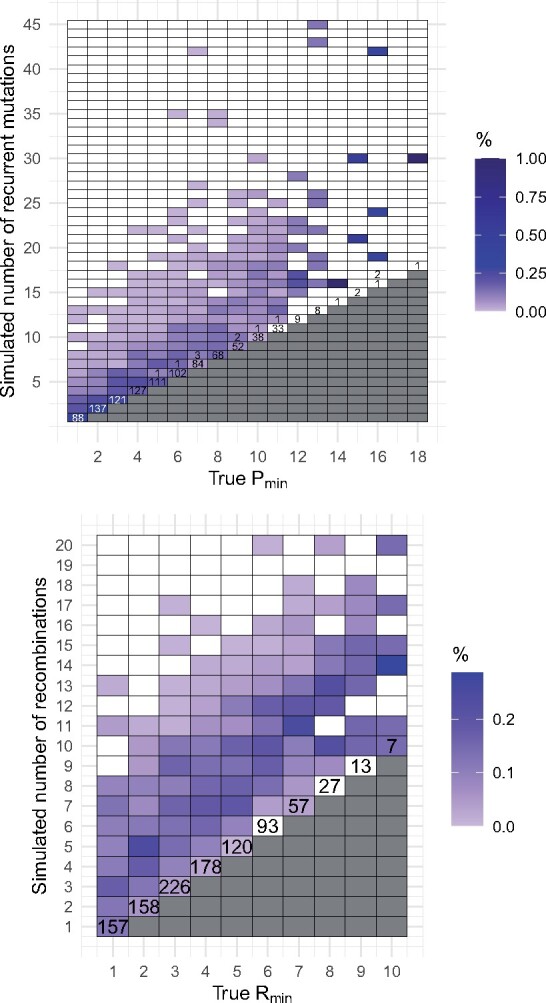
Top: number of simulated recurrent mutations against *P*_min_. Bottom: number of simulated recombinations against *R*_min_. Cell colouring intensity is proportional to the number of datasets generated for each pair of coordinates. Numbers in each cell correspond to the number of cases where for a dataset with the true minimum number of events given on the *x*-axis, KwARG inferred the number of events given on the *y*-axis (unlabelled cells correspond to 0 such cases)

KwARG failed to find *P*_min_ in 11 (1.1%) cases out of 994. The results are illustrated in the top panel of Figure 3. Where KwARG failed to find an optimal solution, in all 11 cases it was off by just one recurrent mutation. Figure 3 also demonstrates that a substantial proportion of recurrent mutations do not create incompatibilities in the data, and the number of actual events often far exceeds *P*_min_.

### 3.2 Infinite sites

#### 3.2.1 Comparison to beagle

Under the infinite sites assumption (disallowing recurrent mutation), the accuracy of KwARG’s upper bound on *R*_min_ was tested by comparison with Beagle (Lyngsø *et al.*, 2005), on 1037 datasets simulated as described in Supplementary Section S3.2.

Using the default annealing parameter *T *=* *30, KwARG found *R*_min_ in all cases. In 97% of the runs, this took under 5 s of CPU time (on a 2.7 GHz Intel Core i7 processor); all but one run took <40 s. In 93% of the runs, one iteration was sufficient to find an optimal solution; in 99% of the runs, five iterations were sufficient. Beagle found the exact solution in 5 s or less in 86% of cases; for datasets with a small *R*_min_ Beagle runs relatively quickly (median run time for *R*_min_ = 5 was 1 s, compared to KwARG’s 0.3 s). For more complex datasets, KwARG finds an optimal solution much faster; for *R*_min_ = 9, the median run time of Beagle was 56 s, compared to KwARG’s 3 s.

Setting *T *=* *10 and T=∞ resulted in 5 and 22 failures to find an optimal solution, respectively, when KwARG was run for Q=1000 iterations per dataset (or terminated after 10 min have elapsed), demonstrating that setting the annealing parameters too low or too high results in deterioration of performance.

The bottom panel of Figure 3 illustrates the results and shows the relationship between the true simulated number of recombinations and *R*_min_. This demonstrates that in many cases, substantially more recombinations have occurred than can be confidently detected from the data.

#### 3.2.2 Comparison to tsinfer, RENT+ and ARGweaver

We tested the performance of KwARG in recovering the topology of simulated local trees for a range of recombination and mutation rates (under the infinite sites assumption). For each combination of rates, we simulated 100 datasets; details of the simulation parameters and settings used in running each program are given in Supplementary Section S5. From the output of each method, we calculated the Kendall–Colijn metric (Kendall and Colijn, 2016) between the inferred and true tree topologies at each variant site position, calculating the mean across all variant sites and averaging over the 100 datasets. We note that ARGs contain more information than local trees, but there is no obvious way of comparing ARG topologies (and tsinfer only infers local trees, rather than full ARGs).

**Fig. 4.**
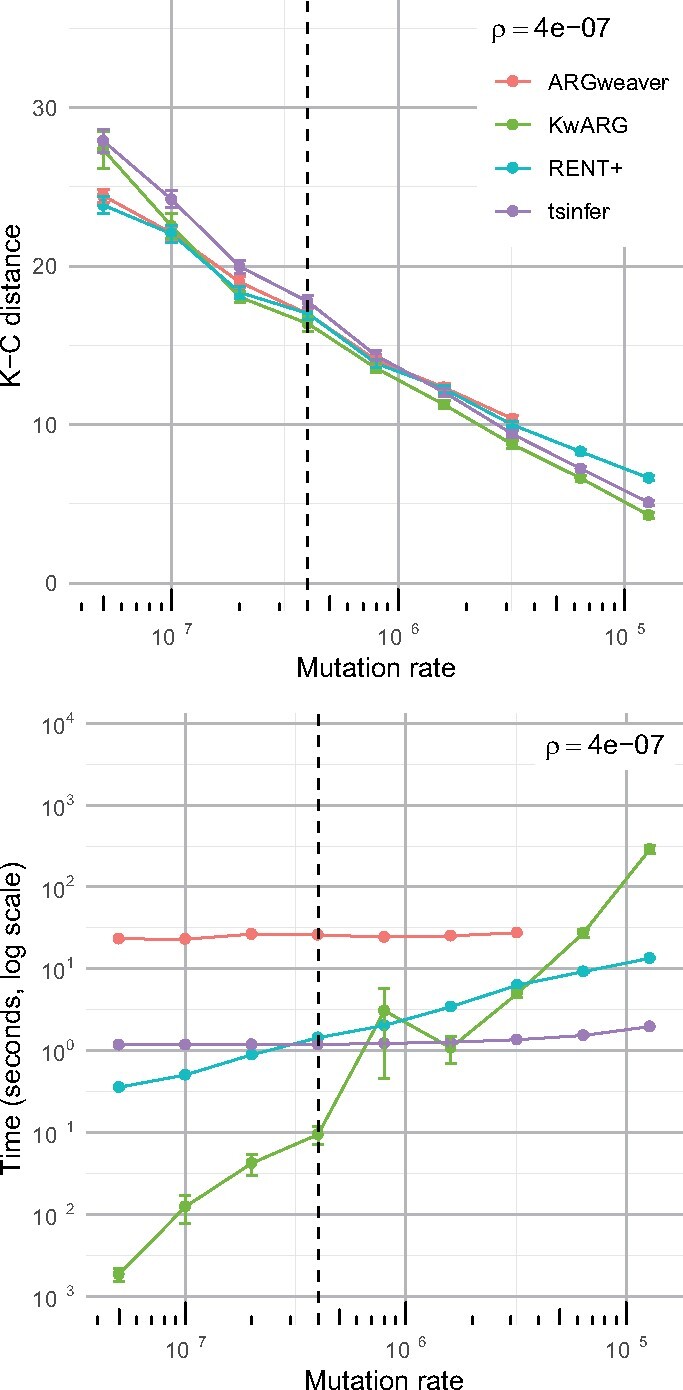
Comparison of performance in inferring local trees. Top panel: points show mean across 100 simulated datasets for each value of mutation rate *μ* (per generation per site) with recombination rate $\rho =4\cdot 10^{-7}$ (per generation per site); error bars show mean ± standard error. Lower K-C distance indicates better accuracy. Bottom panel: points show mean run time averaged over 100 datasets for each combination of rate parameters; error bars show mean ± standard error. ARGweaver results not shown past $\mu =3.2\cdot 10^{-6}$ due to prohibitively long run time

The results are shown in the top panel of Figure 4 and Supplementary Figure S4. All methods show very comparable performance across the range of considered scenarios, with KwARG slightly outperforming the other methods, based on the chosen metric, when the recombination rate is relatively low and the mutation rate relatively high. We have performed the same analysis using the Robinson–Foulds metric (Robinson and Foulds, 1981), and found this to give very similar results.

### 3.3 Run time analysis

A comparison of the run times of KwARG against tsinfer, RENT+ and ARGweaver is presented in the bottom panel of Figure 4 and Supplementary Figure S5. KwARG demonstrates good efficiency when the recombination and mutation rates are relatively low, and shows roughly linear growth in run time as the mutation rate increases.

The dependence of the run time of KwARG on the number and length of sequences was further investigated through simulations; the results are presented in Supplementary Section S6. Keeping the sequence length fixed showed that KwARG runs very quickly when the number of sequences is very low, and shows roughly exponential growth in run time when the number of sequences is 6 or more. Keeping the number of sequences fixed shows that, after an initial exponential increase (due to small datasets taking very little time per iteration), the run time scales roughly linearly in sequence length.

## 3.4 Application to Kreitman data

**Fig. 5.**
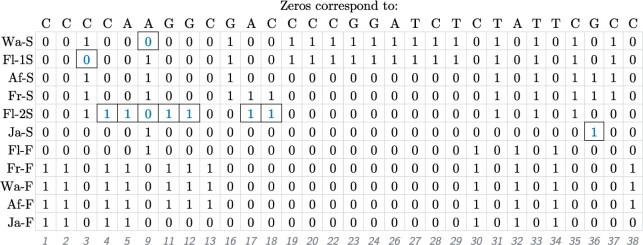
Illustration of the Kreitman dataset. The 11 sequences labelled as in Kreitman (1983); polymorphic sites are labelled 1–43 and columns with singleton mutations are not shown

The performance of KwARG is illustrated on the classic dataset of Kreitman (1983, Table 1); this is not close to the performance limit of KwARG, but has been widely used for benchmarking algorithms used for ARG reconstruction. The dataset consists of 11 sequences and 2721 sites, of which 43 are polymorphic, of the alcohol dehydrogenase locus of *D.melanogaster*. The data are shown in Figure 5, with columns containing singleton mutations removed for ease of viewing. Applying the ‘Clean’ algorithm, as described in Section 2.2.1, reduces this to matrix of 9 rows and 16 columns. KwARG was run with the default parameters, *Q *=* *500 times for each of 13 default cost configurations given in Supplementary Section S2. An example of the output is shown in Table 1.

KwARG correctly identified the *R*_min_ of 7 and the *P*_min_ of 10 (confirmed by running Beagle and PAUP*, respectively). The 6500 iterations of KwARG took just under 9 min to run. Of these, 1829 (28%) resulted in optimal solutions; some are shown in Table 1. KwARG identified multiple combinations of recombinations and recurrent mutations that could have generated this dataset. By default, slightly cheaper costs are assigned to recurrent mutations if they happen on terminal branches, so the results show a bias towards solutions with more *SE* events for each given number of recombinations.

**Fig. 6.**
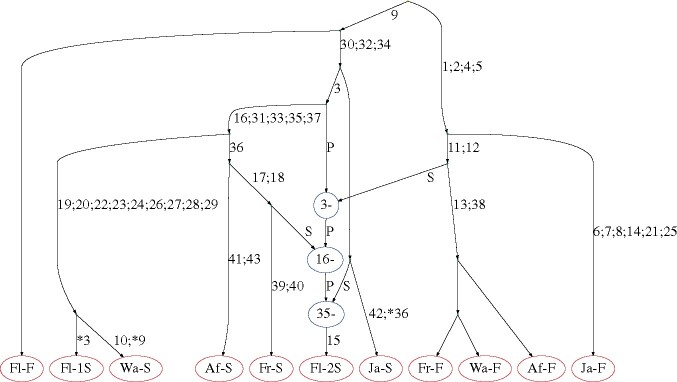
ARG constructed for the Kreitman data. Edges are labelled with sites undergoing mutations; recurrent mutations are prefixed with an asterisk. Recombination nodes, in blue, are labelled with the recombination breakpoint; material to the right (left) of the breakpoint is inherited from the parent connected by the edge labelled *S* (*P*) for ‘suffix’ (‘prefix’)

The ten recurrent mutations appearing in the solution in row 8 of Table 1 are highlighted on the dataset in Figure 5. It is striking that 7 of these 10 recurrent mutations affect the same sequence Fl-2S. In fact, these seven recurrent mutations could be replaced by three recombination events affecting sequence Fl-2S, with breakpoints just after sites 3, 16 and 35; leaving the other identified recurrent mutations unchanged yields the solution in row 5 of Table 1. These findings suggest that the sequence may have been affected by cross-contamination or other errors during the sequencing process, or it could indeed be a recombinant mosaic of four other sequences in the sample. This recovers the results obtained by Stephens and Nei (1985), who posited the recombinant origins of sequence Fl-2S following manual examination of a reconstructed maximum parsimony tree, which also highlighted the five consecutive mutations identified by KwARG. The ARG corresponding to the solution in row 5 of Table 1, visualized using Graphviz (Ellson *et al.*, 2004), is shown in Figure 6.

Examination of the identified solutions also shows that site 36 of sequence Ja-S ‘necessitates’ two of the seven recombinations inferred in the minimal solution in the absence of recurrent mutation, while sites three and nine in sequences Wa-S and Fl-1S, respectively, each create incompatibilities that could be resolved by one recombination.

## 4 Discussion

Methods for the reconstruction of parsimonious ARGs generally rely on the infinite sites assumption. When examining the output ARGs, it is often difficult to tell by how much the inferred recombination events actually affect the recombining sequences. As is the case with the Kreitman dataset, sometimes further examination reveals that two crossover recombination events have the same effect as one recurrent mutation, raising questions about which version of events is more likely. KwARG removes the need for such manual examination, and provides an automated way of highlighting such cases, which is particularly useful for larger datasets.

While KwARG performs well in inferring ARGs under the infinite sites assumption, it can be particularly useful in analysing genetic data from organisms whose genomes are reasonably likely to undergo recurrent mutation, such as viruses with relatively high mutation rates and short genomes. One such application is demonstrated in Ignatieva *et al.* (2021), where the output of KwARG is combined with probabilistic arguments to investigate the presence of ongoing recombination in SARS-CoV-2.

The solutions identified by KwARG differ in the proportion of recurrent mutations to recombinations, ranging from an explanation that invokes only recombination events to one that invokes only mutation events. As is the case with other heuristic and parsimony-based methods, KwARG cannot offer uncertainty quantification for the inferred ARGs. Quantifying the likelihood of each scenario will be application-specific; for instance, one can choose a reasonable model of evolution for the population being studied, and identify the most likely solution under a range of reasonable mutation and recombination rates. When the presence or absence of recombination is not certain, then should the number of recurrent mutations needed to explain the dataset be infeasibly large, this provides evidence for the presence of recombination; this is the idea underlying the homoplasy test of Maynard Smith and Smith (1998). If the largest ‘reasonable’ number of recurrent mutations is then estimated, KwARG can be used to say how many additional recombination events are required to explain the dataset.

KwARG performs well when compared against exact parsimony methods for the ‘recombination-only’ and ‘mutation-only’ scenarios. Because of the random exploration incorporated within KwARG, it should be run multiple times on the same dataset before selecting the best solutions; the optimal run length of KwARG will be constrained by timing and the available computational resources. To gauge whether KwARG has run enough iterations, one could proceed by calculating *R*_min_ and *P*_min_ either exactly (if the data is reasonably small) or using other heuristics-based methods (such as SHRUB or PAUP*), to confirm whether KwARG has found good solutions at these two extremes.

The range of solutions explored by KwARG is guided by the choice of cost parameters. As a rule of thumb, simulations have shown that if the mutation and recombination rates are similar, costs near one give good accuracy of solutions in terms of reconstructing local tree topologies; if the mutation rate is significantly higher (lower) than the recombination rate, the cost should be set to less than (greater than) one. As KwARG incorporates a degree of random exploration, a range of solutions will still be obtained; the best choice of parameters will depend strongly on the nature and aims of the analysis being performed.

For model-based inference, the modelling assumptions can obviously affect the quality of the results; however, a parsimony-based approach also makes the strong assumption that the minimal ARG can capture useful information about the history of a sample. This will obviously depend strongly on the true recombination rate. Based on our comparisons with RENT+, tsinfer and ARGweaver, KwARG achieves very good accuracy of inference of local tree topologies at least comparable to these other methods, particularly when the recombination rate is low to moderate and the mutation rate moderate to high. We emphasize that KwARG demonstrates relatively good accuracy even when the recombination rate is high and even though its express goal is to seek the most parsimonious, rather than necessarily the most likely, history. Moreover, for datasets with relatively few incompatibilities, the run time of KwARG is competitive with that of the other methods. It is also interesting to note that although all four programs incorporate very different approaches and heuristic algorithms, they demonstrate very similar performance in inferring local tree topologies over the range of considered scenarios.

The scalability of KwARG remains a challenge for large and more complex datasets. Performance gains could be readily achieved by running multiple iterations of KwARG in parallel, or incorporating more efficient ways of storing the intermediate states. Further improvements could also be obtained by amending the calculation of lower bounds within the cost function in order to account for the presence of recurrent mutation, which should make the scores more accurate, and hence the neighbourhood exploration more efficient. Other avenues for further work include explicitly incorporating gene conversion as a possible type of recombination event with a separate cost parameter, with a view to developing the underlying model of evolution to even more closely reflect biological reality.

## Supplementary Material

btab351_supplementary_data
