# Supplementary material for: KwARG: parsimonious reconstruction of ancestral recombination graphs with recurrent mutation
Source: Bioinformatics. 2021 May 10;37(19):3277–84. doi: 10.1093/bioinformatics/btab351 (PMC8504621; doi:10.1093/bioinformatics/btab351)
Supplement: btab351_supplementary_data [file btab351_supplementary_data.pdf]

KwARG: PARSIMONIOUS RECONSTRUCTION OF ANCESTRAL RECOMBINATION GRAPHS WITH RECURRENT MUTATION

ANASTASIA IGNATIEVA[1], RUNE B. LYNGSØ[2], PAUL A. JENKINS[1 3 4], AND JOTUN HEIN[2 4]

Supplementary Materials

[1] Department of Statistics, University of Warwick, Coventry CV4 7AL, UK
[2] Department of Statistics, University of Oxford, 24-29 St Giles', Oxford OX1 3LB, UK
[3] Department of Computer Science, University of Warwick, Coventry CV4 7AL, UK
[4] The Alan Turing Institute, British Library, London NW1 2DB, UK

S1. KwARG pseudocode

Let $\mathcal{D}$ be an input data matrix with entries 0, 1 or $\star$. Denote by $\mathcal{D}_{i,j}$ the entry of $\mathcal{D}$ at position (i, j). Let $R_r(\mathcal{D}, i)$ and $R_c(\mathcal{D}, j)$ denote the resulting matrix when the i-th row or the j-th column of $\mathcal{D}$ is deleted, respectively. Let the history $\mathcal{H}$ be a set storing all of the intermediate states visited on the path from $\mathcal{D}$ to the root of the ARG.

Algorithm 1: Clean (adapted from Song and Hein, 2003)

Input: Dataset $\mathcal{D}$, history $\mathcal{H}$
Output: Reduced dataset $\overline{\mathcal{D}}$, updated history $\mathcal{H}'$
Initialise $C \leftarrow$ true, $\overline{\mathcal{D}} \leftarrow \mathcal{D}$, $\mathcal{H}' \leftarrow \mathcal{H}$;
while C **do**
  if *two distinct rows i, j agree:* $\overline{\mathcal{D}}_{i,k} \in \{\overline{\mathcal{D}}_{j,k}, \star\}\ \forall k$ **then**
    $\overline{\mathcal{D}} \leftarrow R_r(\overline{\mathcal{D}}, i)$, $\mathcal{H}' \leftarrow \mathcal{H}' \cup \overline{\mathcal{D}}$;
  else if *there is a column i such that* $\overline{\mathcal{D}}_{k,i} = 1$ *for exactly one k* **then**
    $\overline{\mathcal{D}} \leftarrow R_c(\overline{\mathcal{D}}, i)$, $\mathcal{H}' \leftarrow \mathcal{H}' \cup \overline{\mathcal{D}}$;
  else if *two distinct neighbouring columns i, j agree:* $\overline{\mathcal{D}}_{k,i} \in \{\overline{\mathcal{D}}_{k,j}, \star\}\ \forall k$ **then**
    $\overline{\mathcal{D}} \leftarrow R_c(\overline{\mathcal{D}}, i)$, $\mathcal{H}' \leftarrow \mathcal{H}' \cup \overline{\mathcal{D}}$;
  else
    $C \leftarrow$ false;
end
return $(\overline{\mathcal{D}}, \mathcal{H}')$;

Define the following operations:

(1) Recurrent mutation: $\widetilde{\mathcal{D}} = \mathrm{RM}(\mathcal{D}, i, j)$ is the result of a recurrent mutation in row i at column j; $\widetilde{\mathcal{D}}$ is obtained from $\mathcal{D}$ by changing the (i, j)-th entry from 0 to 1 or from 1 to 0.
(2) Recombination: $\widetilde{\mathcal{D}} = \mathrm{Rec}(\mathcal{D}, i, j)$ is the result of a recombination in row i with breakpoint just after column j. Namely, $\widetilde{\mathcal{D}}$ is obtained from $\mathcal{D}$ by inserting a copy of the i-th row just below itself, and setting $\widetilde{\mathcal{D}}_{i,k} = \star\ \forall k \leq j$ and $\widetilde{\mathcal{D}}_{i+1,k} = \star\ \forall k > j$.
(3) Two consecutive recombinations: $\widetilde{\mathcal{D}} = \mathrm{RRec}(\mathcal{D}, i, j, k, l)$ is the result of performing two recombinations, in rows i and k with breakpoints at j and l, respectively.

Note that for recombination events, not all row and column positions should to be considered, as some moves are guaranteed not to resolve any incompatibilities in the dataset. We apply the ideas detailed in Lyngsø et al. (2005, Section 3.3) to restrict the rows and breakpoints considered for recombination events. Suppose that as a result, $\mathcal{R}$ is the list of row and column indices (i, j) to consider for recombination events, and $\mathcal{RR}$ is the list of indices (i, j, k, l) to consider for two consecutive recombination events.

Algorithm 2: Neighbourhood

Input: Dataset $\mathcal{D}$
Output: Neighbourhood $\mathcal{N}$

```
Initialise N ← {∅};
for (i, j) ∈ R do
  | N ← N ∪ Rec(D, i, j);
end
for (i, j, k, l) ∈ RR do
  | N ← N ∪ RRec(D, i, j, k, l);
end
for all rows i do
  | for all columns j such that D_{i,j} ≠ ⋆ do
  |   | N ← N ∪ RM(D, i, j);
  | end
end
return N;
```

Algorithm 3: KwARG

Input: Dataset $\mathcal{D}$
Output: History $\mathcal{H}$

```
Initialise i ← 1, H ← {D}, (D̄_1, H) ← Clean(D, H);
while D̄_i ≠ ∅ do
  | N̄_i ← {∅}, L_i ← {∅}, S ← {∅};
  | N_i ← Neighbourhood(D̄_i) = {N_i^1, N_i^2, ...};
  | for j = 1 to |N_i| do
  |   | (N̄_i^j, L_i^j) ← Clean(N_i^j, H ∪ N_i^j);
  |   | N̄_i ← N̄_i ∪ N̄_i^j, L_i ← L_i ∪ L_i^j;
  |   | S ← S ∪ S̃(N̄_i^j, N_i^j, D̄_i), where S̃(N̄_i^j, N_i^j, D̄_i) is computed using (??);
  | end
  | Randomly draw an index k from {1, ..., |N̄_i|} with probabilities proportional to entries
  | of S;
  | Set D̄_{i+1} ← N̄_i^k, H ← L_i^k;
  | i ← i + 1;
end
return H;
```

S2. Default cost configuration

If the number of iterations $Q > 1$ is specified but no costs are input, KwARG runs each of the following 13 cost configurations Q times:

$$\begin{aligned}(C_{SE}, C_{RM}, C_R, C_{RR}) \in \{&(\infty, \infty, 1.0, 2.0), (1.0, 1.01, 1.0, 2.0), (0.9, 0.91, 1.0, 2.0), (0.8, 0.81, 1.0, 2.0),\\ &(0.7, 0.71, 1.0, 2.0), (0.6, 0.61, 1.0, 2.0), (0.5, 0.51, 1.0, 2.0),\\ &(0.4, 0.41, 1.0, 2.0), (0.3, 0.31, 1.0, 2.0), (0.2, 0.21, 1.0, 2.0),\\ &(0.1, 0.11, 1.0, 2.0), (0.01, 0.02, 1.0, 2.0), (1.0, 1.1, \infty, \infty)\}.\end{aligned}$$

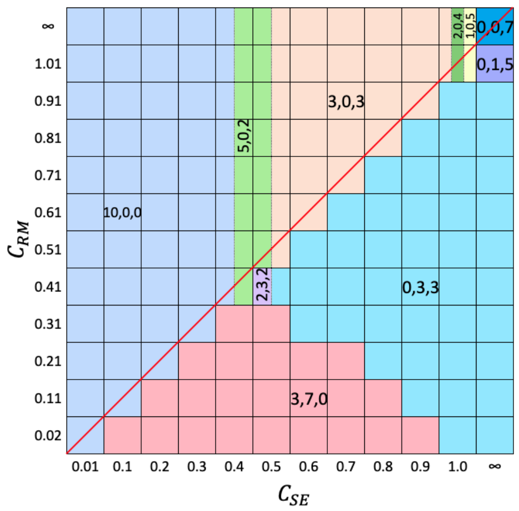

Figure S1. Solution tile plot for the Kreitman dataset.

The effectiveness of this is illustrated in Figure S1, which is based on the set of all possible minimal solutions identified for the Kreitman dataset. Fixing $C_R = 1.0$ and $C_{RR} = 2.0$, each tile represents a pair (C_{SE}, C_{RM}). Each tile is coloured and labelled according to the corresponding cost-optimal solution, in the form $\{x, y, z\}$, giving the number of SE, RM and recombination events, respectively. For instance, if $C_{SE} = 0.5$ and $C_{RM} = 0.61$, the solutions $\{3, 0, 3\}$ (with cost $3 \cdot 0.5 + 3 \cdot 1.0 = 4.5$) and $\{5, 0, 2\}$ (with cost $5 \cdot 0.5 + 2 \cdot 1.0 = 4.5$) have the lowest costs over all feasible solutions.

The default cost configuration includes all pairs (C_{SE}, C_{RM}) on the diagonal in this plot, falling on the red line. This line crosses all optimal solutions which maximise the number of SE events for each possible number of recombinations. Such events affect only a single sequence at a single site in the input dataset, so are, in a sense, more parsimonious than recurrent mutations occurring on internal branches.

S3. Comparison to PAUP* and Beagle

S3.1. **PAUP*.** 1 100 genealogies were simulated using msprime (parameters: 20 sequences, $N_e = 1$). For each tree, Seq-Gen (Rambaut and Grass, 1997) was used to add mutations (parameters: 1 000 sites, mutation rate per generation per site set by the scaling constant $s = 0.01$); only transitions were allowed, to fulfil the requirement that sites mutate between exactly two states. 1 063 datasets exhibited incompatibilities caused by recurrent mutations. KwARG was run for a total of $Q = 600$ iterations per dataset; 150 of these were used to estimate R_{min}, and 450 were run with a range of costs to estimate P_{min}. The runs were terminated after 10 minutes (if 600 iterations had not been completed by then, the results were discarded; this happened in 69 cases); a total of 994 successful runs were performed.

S3.2. **Beagle.** 1 100 datasets were simulated using msprime (Kelleher et al., 2016), under the infinite sites assumption (parameters: $N_e = 1$, mutation rate per generation per site 0.02, recombination rate per site 0.0003, 40 sequences of length 2 000bp). Of the generated datasets, 38 had no incompatible sites, and runs were terminated if Beagle took over 10 minutes to complete (which happened in 25 cases), leaving 1 037 datasets for testing. The parameters were chosen to produce datasets on which Beagle could be run within a reasonable amount of time; the value of R_{min} for the simulated datasets varied between 1 and 10.

S4. Comparison to SHRUB and SHRUB-GC

The performance of KwARG on larger datasets was tested against the parsimony-based heuristic methods SHRUB and SHRUB-GC. Both methods implement a backwards-in-time

construction of ARGs, using a dynamic programming approach to choose among possible recombination events. SHRUB produces an upper bound on R_{min} under the infinite sites assumption. SHRUB-GC also allows gene conversion events; setting the maximum gene conversion tract length to 1 makes this equivalent to recurrent mutation. The algorithm seeks to minimise the total number of events, essentially assigning equal costs to recombination and recurrent mutation. This differs from KwARG in that a single solution is produced for a given dataset, rather than a full range of solutions varying in the number of recombinations and recurrent mutations.

Using msprime and Seq-Gen, 300 datasets of 100 sequences were simulated, with a range of mutation and recombination rates and sequence lengths of 2 000, 5 000, 8 000 and 10 000 bp. For each dataset, KwARG was run for a total of $Q = 260$ iterations, with the default cost configurations and $T = 30$. The resulting upper bound on R_{min} was compared to that produced by SHRUB, and the minimum number of events over all identified solutions was compared to the solution produced by SHRUB-GC (configured to allow length-1 gene conversions).

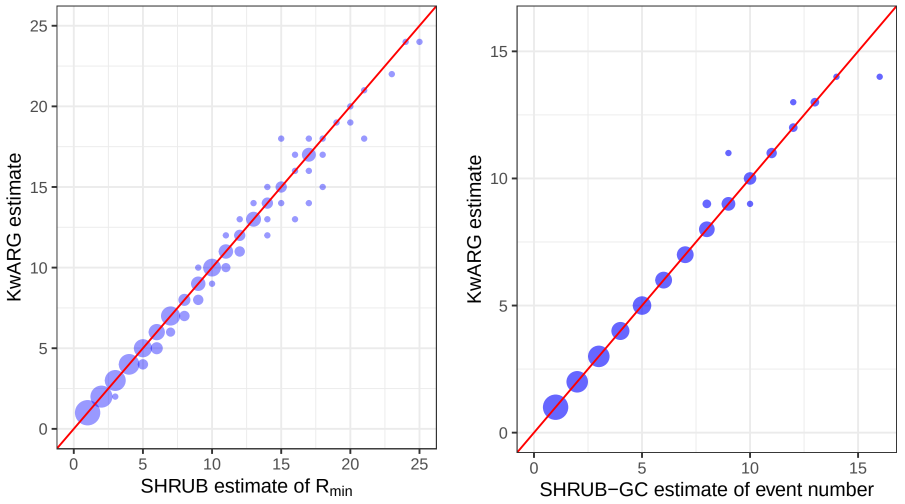

Figure S2. Comparison of KwARG to SHRUB and SHRUB-GC. x-axis: estimate produced by SHRUB (left) and SHRUB-GC (right). y-axis: estimate produced by KwARG. Instances where equally good solutions were found lie on the red diagonal line. Size of points is proportional to the number of corresponding datasets.

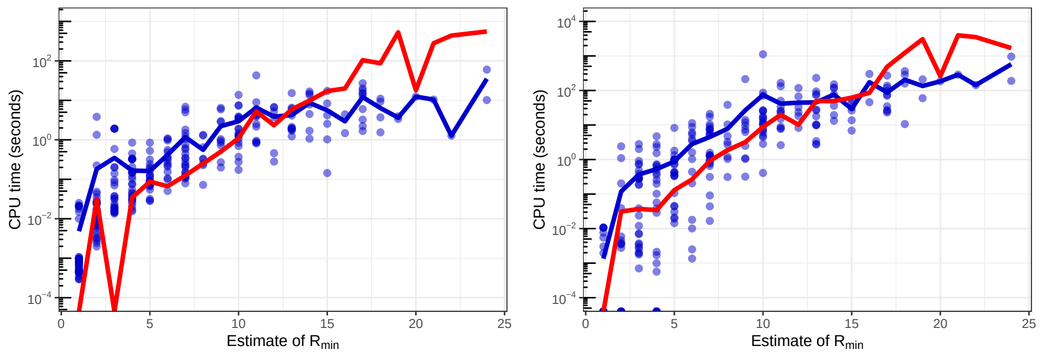

Figure S3. Blue points: time taken to run $Q = 20$ iterations of KwARG (left: disallowing recurrent mutations, right: allowing both recombination and recurrent mutation). Blue lines: mean values. Red line: mean run time of SHRUB (left) and SHRUB-GC (right). Time in seconds is given on a log scale.

KwARG obtained solutions at least as good as SHRUB's in 292 (97.3%) of 300 cases, outperforming it in 35 (11.7%) instances. KwARG obtained solutions at least as good as SHRUB-GC

in 296 (98.7%) cases, outperforming it in 2 instances. The results and the run times are illustrated in Figures S2 and S3. On average, for relatively small and simple datasets, KwARG takes approximately the same time per one iteration as a run of SHRUB or SHRUB-GC, and outperforms both programs on more complex datasets.

S5. Comparison to tsinfer, RENT+, and ARGweaver

Datasets were simulated using msprime under the infinite sites assumption (parameters: $N_e = 10\,000$, 20 sequences of length 1 000bp), with a range of recombination rates ($\{1 \cdot 10^{-7}, 2 \cdot 10^{-7}, 4 \cdot 10^{-7}, 8 \cdot 10^{-7}, 1.6 \cdot 10^{-6}\}$ per site per generation) and mutation rates ($\{5 \cdot 10^{-8}, 1 \cdot 10^{-7}, 2 \cdot 10^{-7}, 4 \cdot 10^{-7}, 8 \cdot 10^{-7}, 1.6 \cdot 10^{-6}, 3.2 \cdot 10^{-6}, 6.4 \cdot 10^{-6}, 1.28 \cdot 10^{-5}\}$ per site per generation). These parameters were chosen to cover a broad range of the simulated number of recombinations and mutations. 100 datasets were simulated for each combination of rates.

RENT+, tsinfer, ARGweaver, and KwARG were run on each dataset. For tsinfer, the ancestral state must be specified at each variable site, and was set to the simulated truth. ARGweaver requires the specification of mutation and recombination rates; these were set to the simulation parameters used. ARGweaver was run for 1 200 iterations, discarding the first 1 000 as burn-in, and then sampling ARGs with intervals of 20 steps (obtaining 10 in total). KwARG was run for one iteration per dataset, with the parameters $T = 30$, $C_{SE} = C_{RM} = \infty$, and the known ancestral sequence set as the root.

For each dataset, the local trees output by each program were then compared to the simulated true trees, by calculating the Kendall–Colijn metric at each variable site position. As tsinfer can output trees with unresolved polytomies, these were resolved randomly before calculating the metric for the sake of fair comparison. The mean was then calculated across sites, and for each combination of recombination and mutation rate the metric was averaged across the datasets. The results are presented in Figure S4. A comparison of the run times of the programs used is illustrated in Figure S5.

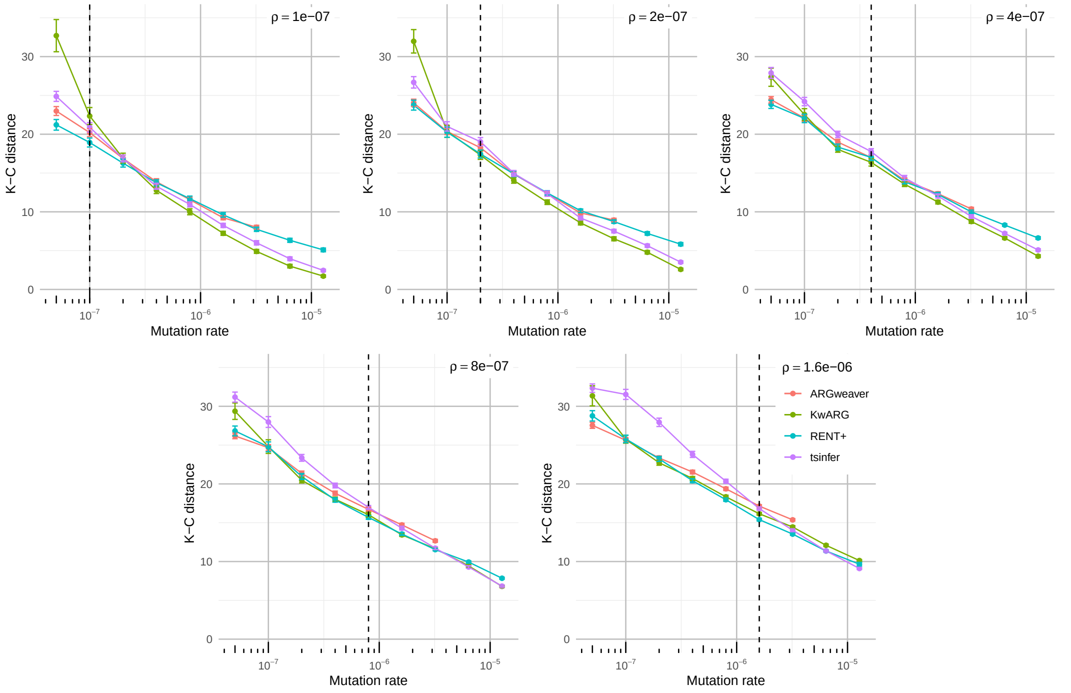

Figure S4. Comparison of performance in local tree recovery. Dashed vertical lines show the value of the recombination rate in each panel. Points correspond to mean values; error bars show mean ± standard error. ARGweaver results not shown past $\mu = 3.2 \cdot 10^{-6}$ due to prohibitively long run time. Lower K-C distance indicates better accuracy.

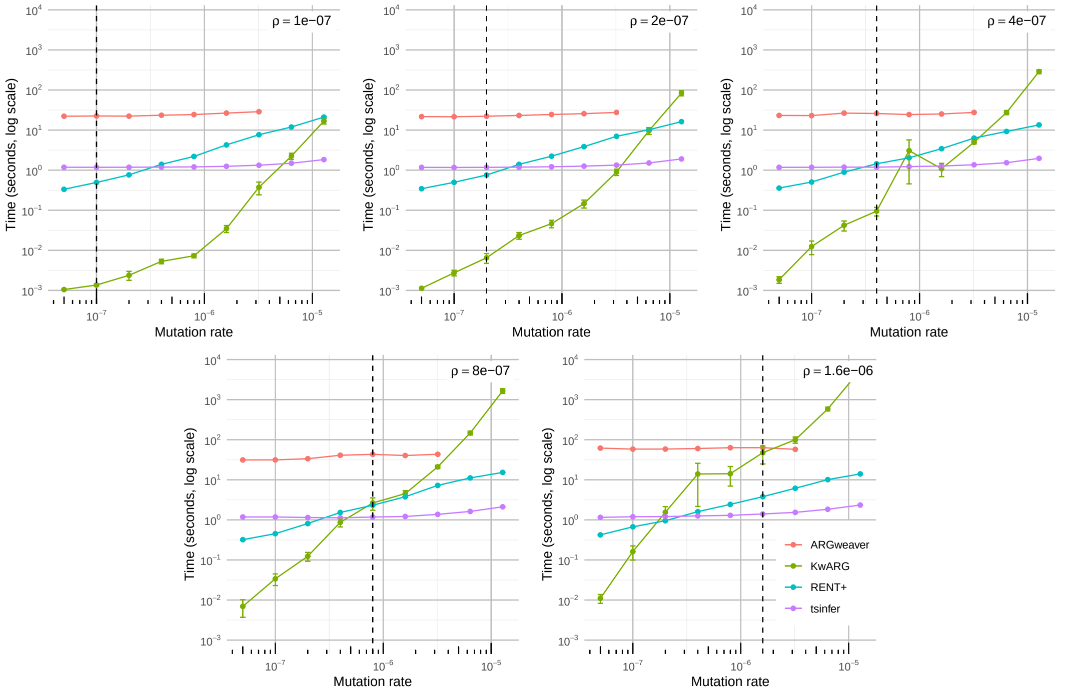

Figure S5. Comparison of time taken per dataset. Points show mean run time averaged over 100 datasets for each combination of rate parameters. Error bars show mean ± standard error.

S6. Time complexity

The scaling of KwARG's run time was investigated through simulation. First, we fixed the sequence length at 5 000bp, and simulated datasets with varying numbers of sequences (from 2 to 30) using msprime, with the infinite sites assumption (parameters: $N_e = 10\,000$, mutation rate $2 \cdot 10^{-7}$ per site per generation, recombination rate $2 \cdot 10^{-7}$ per site per generation). 500 simulations were carried out for each number of sequences; for each dataset, KwARG was run once and the runtime recorded. The results are presented in the left panel of Figure S6. KwARG runs very quickly when the number of sequences is very low, and shows roughly exponential growth in run time when the number of sequences is 6 or more.

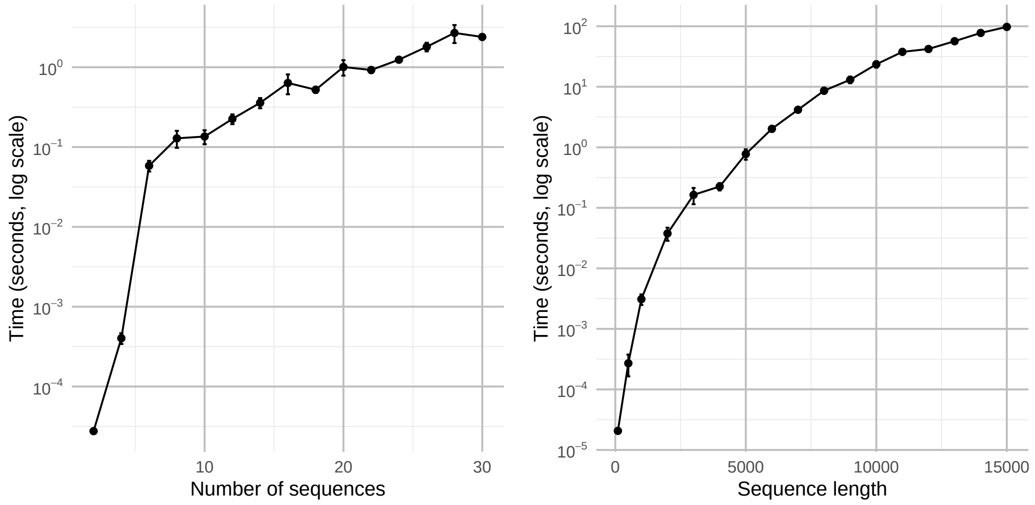

Figure S6. Run time versus number of sequences (left panel) and sequence length (right panel). Lines show mean run time over 500 (100) datasets; error bars show mean ± standard error.

Next, we fixed the number of sequences at 20, and simulated datasets with varying sequence lengths (from 100 to 15 000bp) using msprime, with the infinite sites assumption (same parameters as above). 100 simulations were carried out for each sequence length; for each dataset, KwARG was run once and the runtime recorded. The results are presented in the right panel of Figure S6. After an initial exponential increase (due to small datasets taking very little time per iteration), the run time scales roughly linearly in sequence length.

References

Kelleher, J., Etheridge, A. M. and McVean, G. (2016). Efficient coalescent simulation and genealogical analysis for large sample sizes. *PLoS Computational Biology*, **12**(5), 1–22.

Lyngsø, R. B., Song, Y. S. and Hein, J. (2005). Minimum recombination histories by branch and bound. In *International Workshop on Algorithms in Bioinformatics*, pp. 239–250. Springer.

Rambaut, A. and Grass, N. C. (1997). Seq-Gen: an application for the Monte Carlo simulation of DNA sequence evolution along phylogenetic trees. *Bioinformatics*, **13**(3), 235–238.

Song, Y. S. and Hein, J. (2003). Parsimonious reconstruction of sequence evolution and haplotype blocks. In *International Workshop on Algorithms in Bioinformatics*, pp. 287–302. Springer.
